# The effect of music on menopausal symptoms, sleep quality,and depression: a randomized controlled trial

**DOI:** 10.1590/1806-9282.20230829

**Published:** 2024-01-22

**Authors:** Meltem Ugurlu, Meryem Vural Şahin, Oznur Hayat Oktem

**Affiliations:** 1 University of Health Sciences, Gulhane Faculty of Health Sciences, Department of Midwifery – Ankara, Turkey.; 2 University of Health Sciences, Health Sciences Institute of Gulhane, Department of Midwifery – Ankara, Turkey.

**Keywords:** Sleep quality, Depression, Menopause, Music, Randomized controlled trial

## Abstract

**OBJECTIVE:**

This study aimed to determine the effect of music on menopausal symptoms, sleep quality, and depression levels in menopausal women.

**METHODS:**

This randomized controlled study was carried out between August and December 2022. The study sample consisted of 61 menopausal women (intervention: 30 and control: 31). The intervention group listened to music twice a day for 5 weeks, with a total of 70 sessions. The control group received only routine care. Menopause symptoms, depression levels, and sleep quality were evaluated at the beginning and the end of the study using the Menopausal Symptoms Rating Scale, Beck Depression Inventory, and Pittsburg Sleep Quality Index.

**RESULTS:**

The post-test Menopausal Symptoms Rating Scale, Beck Depression Inventory, and Pittsburg Sleep Quality Index scores of the menopausal women were found to be lower in the intervention group than in the control group (p=0.011, p=0.001, and p=0.006, respectively). When the pre-test and post-test mean scores were compared, the mean menopausal symptoms and depression levels decreased, and sleep quality increased significantly in the intervention group. No significant difference was observed in the control group.

**CONCLUSION:**

This study shows that music may have an effect on reducing the level of menopausal symptoms and depression levels and also increasing the sleep quality of menopausal women.

## INTRODUCTION

Menopause is an important turning point in the life cycle of women^1^. Menopausal women complain about numerous different symptoms such as sexual problems, night sweats, vaginal dryness, sleep problems, as well as psychological changes such as depression. Approximately 70% of women complain about vasomotor symptoms such as hot flashes, which may continue for up to 15 years in about 20% of women^2-4^. Another common complaint related to menopause is sleep disorders, which is an important global health problem affecting 14.3–53.6% of women^5,6^. Poor sleep quality is associated with negative health outcomes such as obesity, poor quality of life, and depression^6^. In addition, as the risk of developing depressive symptoms due to poor sleep quality may increase 2–3 times^5^, there is a vicious circle between menopausal symptoms, sleep quality, and depression. Midwives and nurses can contribute to the reduction of menopause symptoms/depression and increase the sleep quality by executing the roles of counseling, education, and care during menopause^3^.

In recent years, the trend toward non-drug methods such as reflexology, acupuncture, and yoga has increased in curing the problems caused by menopausal symptoms^4,7^. Music intervention is one of the useful, easy-to-use, and accessible alternative methods. Music can help improve sleep quality by affecting the limbic system of the brain^8^. While endorphin, serotonin, dopamine, and oxytocin hormones increase with the effect of music, the levels of stress hormones such as cortisol decrease, and under the influence of hormones, breathing slows down; blood pressure drops; blood vessels expand; and muscle relaxation accelerates. Thus, music creates a relaxation effect that reduces anxiety and stress and increases sleep quality^8,9^. Several studies determined that music was effective in reducing depression^10^ and improving sleep quality^10,11^ in adults. However, only one study focused on the effect of music in menopausal women but did not evaluate its effect on sleep quality^3^. Therefore, the results of this study will make an important contribution to the literature with data from a developing country. This study was performed to investigate the effect of music provided for 5 weeks on menopausal symptoms, depression levels, and sleep quality in menopausal women.

## METHODS

### Study design and location

This study was a randomized-controlled study with a pre-test-post-test design. The study was conducted between August 15 and December 25, 2022, in a family medicine located in the central Anatolia region of Turkey. The study was conducted in accordance with CONSORT guidelines and registered on clinicaltrials.gov (registration no. NCT05545761). The suggestions developed by Robb et al.^12^ were used in reporting the music intervention.^k^

### Sample size and characteristics

The sample size was determined based on a similar study in the literature^13^. The sample was calculated using the G Power 3.1.9.7 program. With 95% confidence (1–α), 90% test power (1–β), d=0.89 effect size, and two-way t-test, the minimum sample size was determined as 29 women in each group. Considering possible data loss, a total of 70 menopausal women with 35 in each group were included in the study. Five women in the intervention group and four in the control group dropped out. As a result, with 30 cases in the intervention group and 31 cases in the control group, a post-hoc power analysis was performed, and the power of the test was calculated as 91% (1–β) with 95% confidence interval (1–α) and an effect size of d=0.89. It was determined that the sample size was sufficient for the study. Women in menopause aged between 40 and 65 years, who had not been diagnosed with any psychiatric disease, had no hormone therapy for menopause, had no medical treatment for sleep problems, and had no hearing, mental, or communication problems were included in the study. Those who did not meet the inclusion criteria were excluded from the study.

### Data collection tools

The questionnaire consisted of four parts. The first part designed to extract information about menopausal women’s sociodemographic characteristics (e.g., age, age of menopause, educational status, working status, level of income, presence of chronic disease, and smoking habits) prepared by the researchers in line with the literature^5,6,9,13^ consists of 7 items.

In the second part, the MRS evaluates the menopausal symptoms and the severity of these symptoms. The Turkish validity and reliability study of the scale was conducted by Metintas et al.^14^. The five-point Likert-type scale consists of 11 items and they are grouped under three factors: somatic, psychological, and urogenital complaints. The total score obtained from the scale varies between 0 and 44. Higher total mean scores indicate greater severity of menopausal symptoms. In the third part of the questionnaire, the BDI evaluates the physical, emotional, and cognitive symptoms of depression and consists of 21 items, with a scoring system between 0 and 3 points^15^. The total score varies between 0 and 63. An increase in the total score indicates an increase in depression. The Turkish form of the PSQI was used to assess sleep quality^16^. It is calculated over 19 items and scored between 0 and 3. The total PSQI score ranges from 0 to 21. A PSQI score of 5 and above is considered “poor sleep quality,” and a score of 5 or below is considered “good sleep quality.”

### Randomization

For randomization, the participants were assigned to the groups by the researchers using the Random Integer Generator (www.random.org, date: 05.08.2022).

### Data collection

After the consent of all menopausal women was obtained, and randomization was performed, the sociodemographic, menopausal symptoms, depression, and sleep quality levels were determined via the pre-test. Both groups continued to receive routine care during the study period

### Control group

This group only received routine care in the family medicine. Five weeks after the pre-test (introductory information form, MRS, BDI, and PSQI), the post-test (MRS, BDI, and PSQI) was applied.

### Intervention group

After the pre-test, the intervention group listened to music for two sessions (in the morning: Hüseyni maqam; at night: Zirgüle maqam) in a day for 5 weeks, selected by the researchers. The Hüseyni maqam, which is most effective between morning and noon, was used for the effects of giving people calmness and comfort by emphasizing beauty and goodness. The Zirgüle maqam was used for the effects of improving sleep to people. Participants were contacted via WhatsApp and were ensured that they were listening to music. The pieces of music were accessed free of charge from the YouTube website and sent to the women. The participants listened to the selected music pieces in their own homes, in a quiet environment, and with headphones. The volume of the music was determined by the women. In total, 70 music sessions were conducted with 30 menopausal women. The average time for listening to music was 30 min (15 min each morning and night) for each day. Post-test was applied to the intervention group when music listening was terminated after 5 weeks.

### Evaluation of data

Chi-square-based hypothesis test was applied to test the relationship between categorical variables. We used chi-square independence tests and Fisher’s exact tests for nominal variables. We carried out an independent-samples t-test and Mann-Whitney U tests to compare the MRS, BDI, and PSQI scores between the intervention and control groups. Also, we implemented a paired-samples t-test and Wilcoxon test to compare the pre-post MRS, BDI, and PSQI scores for each intervention group and control group.

### Ethics

Ethical permission (decision no: 2022/1017, date: 04.07.2022) and necessary permissions were obtained. The research was conducted in accordance with the Declaration of Helsinki.

## RESULTS

The sociodemographic characteristics of the participants are shown in Table 1, and there is no significant difference between the groups. Participants’ age, age of menopause, educational status, working status, level of income, chronic disease state, and smoking habits are similar (p>0.05).

**Table 1.** Sociodemographic characteristics between the groups.

	Intervention group (n=30)	Control group (n=31)	p-value
Age	53.40 (4.75)	52.48 (5.92)	0.443^U^
Age of menopause	48.43 (4.14)	47.35 (4.16)	0.202^U^
Educational status			
Primary school	4 (13.3)	12 (38.7)	0.070^F^
High school	17 (56.7)	13 (41.9)	
University and above	9 (30.0)	6 (19.4)	
Working status			
Not working	18 (60)	23 (74.2)	0.364^F^
Working	12 (40)	8 (25.8)	
Level of income			
Low	3 (10)	3 (9.7)	0.997^F^
Moderate	22 (73.3)	23 (74.2)	
High	5 (16.7)	5 (16.1)	
Presence of a chronic disease			
Yes	17 (56.7)	17 (54.8)	0.886^F^
No	13 (43.3)	14 (45.2)	
Smoking habit			
I have never smoked	17 (56.7)	20 (64.5)	0.799^F^
I do not smoke now	3 (10.0)	3 (9.7)	
I am a smoker	10 (33.3)	8 (25.8)	

Categorical variables are presented as n (%) and continuous variables as mean (SD).

U Mann-Whitney U test;

F Fisher’s exact test.

Table 2 shows the comparison of the MRS total and subdimension scores between and within groups. Examination of the inter-group differences revealed that there was no significant difference between the MRS total and subdimension pre-test scores of the intervention and control groups (p>0.05). Post-test MRS total, somatic, and psychological symptoms scores were significantly different between the intervention and control groups (p<0.05). However, no significant difference was found between the urogenital symptom post-test scores (p>0.05).

**Table 2.** Comparison of the Menopause Symptom Rating Scale total and subdimensions scores between and within groups.

MRS total	Intervention group (n=30)	Control group (n=31)	Between groups p-value
Pre-test	15.97 (5.26)	14.74 (5.11)	0.360^IT^
Post-test	12.40 (6.51)	16.00 (4.12)	0.011^IT^
Within group p-value	<0.001^PST^	0.076^PST^	
Somatic symptoms			
Pre-test	6.23 (2.14)	5.39 (1.93)	0.336^U^
Post-test	4.97 (2.13)	5.87 (1.69)	0.006^U^
Within group p-value	0.002^W^	0.146^PST^	
Psychological symptoms			
Pre-test	6.10 (4.57)	6.58 (3.24)	0.586^U^
Post-test	4.57 (3.41)	7.16 (2.71)	<0.001^U^
Within group p-value	0.008^W^	0.130^PST^	
Urogenital symptoms			
Pre-test	3.63 (1.90)	2.77 (2.17)	0.126^U^
Post-test	2.87 (2.13)	3.06 (2.13)	0.604^U^
Within group p-value	0.019^W^	0.239^PST^	

IT Independent-samples t-test;

PST Paired-samples t-test;

U Mann-Whitney U test;

W Wilcoxon test.

Table 3 shows the comparison of the BDI and PSQI scores between and within groups. Examination of the inter-group differences revealed that there was no significant difference between the BDI and PSQI pre-test scores of the intervention and control groups (p>0.05). Post-test BDI and PSQI scores were significantly different between the intervention and control groups (p<0.05). Accordingly, depression levels were lower and sleep quality was higher in the intervention group than in the control group.

**Table 3.** Comparison of the Beck Depression Inventory and Pittsburg Sleep Quality Index scores between and within groups.

BDI	Intervention group (n=30)	Control group (n=31)	Between groups p-value
Pre-test	13.13 (4.07)	12.00 (8.14)	0.158^U^
Post-test	7.50 (4.33)	12.77 (6.82)	0.001^U^
Within group p-value	<0.001^W^	0.084^W^	
PSQI			
Pre-test	9.43 (2.16)	8.29 (2.16)	0.062^U^
Post-test	6.23 (2.58)	8.16 (2.55)	0.006^U^
Within group p-value	<0.001^W^	0.639^W^	

U Mann-Whitney U test;

W Wilcoxon test.

## DISCUSSION

The menopausal period, which begins with the cessation of a woman’s reproductive phase, is an important transitional period involving significant physical and emotional changes. During this period, hypoestrogenism affects the central nervous system and triggers common symptoms such as hot flashes, sweating, and insomnia, which can impact women’s activities and quality of life^17,18^. According to the results of the study, music provides a significant improvement in the sleep quality of menopausal women. The meta-analysis reported that music helped to improve the sleep quality of patients with acute and chronic sleep disorders^19^. It was determined that music-based interventions, which are safe and easy to apply, can effectively improve sleep quality among older adults who listen to soothing music for at least 4 weeks^5^. The results of studies conducted with students, pregnant women, and burn patients showed that music significantly increased sleep quality^20-22^. Another randomized controlled study revealed that music applied for 4 weeks provided significant improvements in sleep quality in adults with depression, and the improvement in sleep quality scores decreased 4 weeks after the end of the music intervention. Music has been suggested as a safe and effective sleep aid in insomnia associated with depression^11^. This result showed that a 5-week music intervention is an effective method of increasing sleep quality in menopausal women.

Our study revealed that the menopause symptoms of the participants in the intervention group decreased significantly. Similar results were obtained in a recent study conducted with menopausal women in Turkey^3^. In a study, a music-based program designed to teach brisk breathing to reduce hot flashes had no effect on hot flashes^23^. A meta-analysis study that investigated the effects of meditation, mindfulness, and relaxation to treat vasomotor symptoms reported that brisk breathing did not have a significant effect on the frequency and severity of vasomotor symptoms, and that various relaxation and mindfulness interventions had no effect on vasomotor symptoms^24^. A review study that examined the effects of non-pharmacological therapies in the management of vasomotor symptoms of menopause in breast cancer survivors stated that cognitive behavioral therapies reduce the perceived effect of hot flashes and night sweats, and yoga and acupuncture can reduce the frequency of vasomotor symptoms^25^. This study showed that music intervention for 5 weeks was effective in reducing menopausal symptoms.

The menopausal transition is a critical period when women are more sensitive to mood changes and the onset of depressive symptoms^26^. Treating sleep disorders, vasomotor symptoms, and urogenital symptoms not only alleviates these issues but also has a positive impact on mood and cognitive symptoms^26^. A meta-analysis study highlights that depression is quite common in menopausal women and underlines the importance of developing effective treatments^1^. Deshpande et al., reported that step aerobics, music, or both can be equally effective in improving the mental health of postmenopausal women^25^. Mo et al., found that classical music therapy 30 min before going to bed for 4 weeks was significantly effective in relieving depression in peri-menopausal women^27^. A meta-analysis found that music therapy was significantly effective in reducing depressive symptoms in adults with depression^28^. Our study found that music intervention applied to women in the menopausal period was effective in reducing depression levels.

Considering that women spend approximately one-third of their lives in the menopausal period due to increased life expectancy, non-pharmacological and side-effect-free methods such as music can be considered an alternative approach to traditional treatments for reducing somatic, psychological, and urogenital symptoms, sleep problems, and depression in menopausal women^18,26^. Healthcare professionals who support women during the menopausal period can offer personalized lifestyle changes, such as incorporating music, as an effective coping strategy through a multidisciplinary team approach^17,26^. In today’s world, the use of digital health is transforming the healthcare provider–patient relationship by enhancing clinical practices^29^. In this context, our study is highly important as it demonstrates that the utilization of technological advancements such as mobile applications and Internet connectivity assists in achieving positive health outcomes by helping to alleviate issues during the menopausal period.

### Limitations

First, the results cannot be generalized to all menopausal women owing to the single-centered nature of the study. Second, the participants were asked to listen to the music with a headset. Some of the participants stated that they were uncomfortable using headphones. This may have affected our results.

## CONCLUSION

Based on these findings, it can be stated that the music intervention applied to menopausal women reduced their depression levels and menopausal symptoms and increased their sleep quality. In this context, it is recommended to increase the awareness of health professionals working in the field of women’s health, especially nurses, about music intervention. In addition, considering the advantages of music such as low cost and no side effects, it is thought that it should be offered routinely, especially within the scope of nursing care interventions. Future studies may investigate the long-term effects of music and evaluate music genres making a comparison between them.

## ETHICAL APPROVAL

Ethics committee permission was obtained from the Karabuk University Ethical Committee with the number 2022/1017 and the date 04.07.2022.
